# Social media social comparison orientation and physical self-efficacy among Chinese college students: sequential mediation via body shame and body appreciation

**DOI:** 10.3389/fpsyg.2026.1823002

**Published:** 2026-05-01

**Authors:** Quan Zhang, Zixuan Yang, Xianlin Xiao, Xingjun Zhou, Yuanye Hu

**Affiliations:** 1 Pai Chai University, Daejeon, Republic of Korea; 2 Faculty of Management, Shinawatra University, Pathum Thani, Thailand

**Keywords:** body appreciation, body shame, college students, physical self-efficacy, sequential mediation, social comparison orientation, social media

## Abstract

**Purpose:**

This study examined the association between social media social comparison orientation and physical self-efficacy among Chinese college students, and tested whether body shame and body appreciation mediated this association independently and sequentially.

**Methods:**

A cross-sectional online survey was conducted among 3,401 undergraduate students from 12 universities in Shandong Province, China. Using convenience sampling, participants were recruited through university-affiliated online channels, including class communication groups, university social media groups, student organization networks, and learning platforms, with dissemination support from local collaborators. Measures of social media social comparison orientation, body shame, body appreciation, and physical self-efficacy were administered in Chinese. Structural equation modeling and bias-corrected bootstrapping were used to estimate direct and indirect associations.

**Results:**

Social media social comparison orientation was negatively associated with physical self-efficacy (β = −0.30) and body appreciation (β = −0.26), and positively associated with body shame (β = 0.32). Body shame was negatively associated with physical self-efficacy (β = −0.19) and body appreciation (β = −0.28), whereas body appreciation was positively associated with physical self-efficacy (β = 0.23; all ps < .001). Bootstrapping indicated a significant total association between social media social comparison orientation and physical self-efficacy [β = −0.441, 95% CI (−0.478, −0.403)], including a significant direct association [β = −0.298, 95% CI (−0.342, −0.253)] and a significant total indirect association [β = −0.143, 95% CI (−0.163, −0.124)]. Specific indirect associations were significant via body shame [β = −0.061, 95% CI (−0.076, −0.049)] and body appreciation [β = −0.061, 95% CI (−0.075, −0.048)]. The sequential indirect association through body shame and body appreciation was also significant [β = −0.021, 95% CI (−0.027, −0.016)].

**Conclusion:**

Higher social media social comparison orientation was associated with lower physical self-efficacy among Chinese college students. Body shame and body appreciation were linked to this association both as separate indirect pathways and as a sequential indirect pathway. These findings underscore the importance of body-related self-conscious emotion and positive body image in understanding how social media comparison orientation relates to perceived physical ability in a Chinese college context.

## 1 Introduction

Social networking platforms have become deeply embedded in the daily lives of college students, providing continuous access to peers' curated images, achievements, and lifestyle narratives. Contemporary social media environments are also increasingly shaped by influencer-centered and highly curated content dynamics, which may further intensify users' exposure to aspirational self-presentations (Fauzi et al., 2024). Such environments intensify opportunities for social comparison, particularly comparisons centered on attractiveness (Fardouly et al., 2015), fitness (Pasko and Arigo, 2021), and perceived competence (de Brabandere et al., 2025). Rather than reflecting occasional curiosity, comparison can operate as a relatively stable orientation that shapes attention allocation (Yang and Robinson, 2018), information processing (Murray-Gibbons and Gibbons, 2023), and self-evaluation (Vogel et al., 2015) during social media browsing. When comparison cues are frequent and highly standardized, subjective self-worth and perceived capability may become increasingly contingent on one's perceived standing relative to others (De Vries and Kühne, 2015; Midgley et al., 2021).

Recent review-level evidence further underscores the importance of examining social media-related comparison processes in the context of body-related outcomes and health. (Bonfanti et al. 2025), in a systematic review and meta-analysis, reported that social comparison on social media is consistently associated with body image concerns and eating disorder symptoms. Similarly, (Sarda et al. 2025) highlighted the roles of self-objectification, self-compassion, and body image concerns in explaining how social media use may shape self-perception and well-being. Extending beyond body-image-specific outcomes, (Behera et al. 2025) synthesized evidence from 21 countries and concluded that social media use is linked to a broad range of physical, mental, social, and emotional health indicators, including body image, mood, and sleep quality. Taken together, these reviews suggest that social media-related comparison is embedded in a broader body image and health framework. At the same time, review-level evidence has focused primarily on body image concerns, eating-related symptoms, and general well-being, with comparatively less attention to capability-related outcomes such as physical self-efficacy, especially in models that simultaneously consider both vulnerability processes and protective body image processes.

The Chinese context warrants explicit consideration in this line of research. China is not merely the location from which the present sample was drawn, but a theoretically meaningful setting in which to examine the comparison–body image–capability linkage. Official statistics indicate that social networking, instant messaging, and short-video use have reached near-universal levels among Chinese internet users (China Internet Network Information Center, 2025), suggesting that Chinese college students navigate a highly saturated and visually intensive digital environment in everyday life. At the same time, prior cross-cultural research suggests that Chinese/Asian and Western media influences should not be treated as interchangeable with respect to body image disturbance (Jackson et al., 2016). Consistent with this point, studies in Chinese samples have shown that media- and peer-related influences are associated with negative body image through appearance comparison and thin-ideal internalization (Shen et al., 2022), that body talk on social networking sites is linked to body dissatisfaction among college students (Wang et al., 2022), and that online media exposure and weight- and fitness-management app use are related to disordered eating symptoms in mainland China (Guo et al., 2022). Accordingly, the present study proceeds from a qualified position: although the basic psychological processes underlying social media comparison may be broadly relevant across regions under conditions of platform globalization, their salience, configuration, and implications should be examined directly in Chinese college students rather than inferred from Western findings alone.

Against this broader and culturally situated background, physical self-efficacy represents a psychologically consequential outcome in the social media context (Hu et al., 2025). Confidence in physical capability influences engagement in physical activity, persistence under fatigue, approach toward performance challenges, and willingness to adopt health-promoting routines (Kurata et al., 2023; Lee et al., 2024). In college populations, physical self-efficacy also carries broader developmental significance. The college years often coincide with greater autonomy over lifestyle choices, fluctuating activity patterns, and heightened sensitivity to appearance norms (Pasko and Arigo, 2021). A systematic erosion of perceived physical ability during this period may constrain participation in exercise, amplify avoidance of evaluative settings such as gyms or sports classes, and reduce resilience following setbacks. Despite a growing body of research linking social media experiences to well-being, the pathway from social media social comparison orientation to physical self-efficacy remains under-specified, particularly in models that integrate both risk and protective processes within body-related self-perception (Le Blanc-Brillon et al., 2025; Wang et al., 2026).

Body-related self-conscious emotions offer one plausible psychological conduit. Body shame captures the distress associated with perceiving oneself as falling short of internalized appearance ideals and attributing that discrepancy to personal inadequacy (Mills et al., 2022). Social media comparison environments heighten exposure to idealized bodies, edited images, and performance-optimized representations, creating conditions that may evoke shame through upward comparison and perceived inadequacy (Guo et al., 2026; Kidd et al., 2024; Portingale et al., 2024; Wang et al., 2025). Body shame is more than transient discomfort; it may motivate concealment, avoidance, and disengagement from physical domains in which the body becomes salient (Kim, 2022). These dynamics are especially relevant for physical self-efficacy, because shame may bias self-appraisal toward deficiencies and undermine confidence in bodily competence (Li et al., 2025).

A more complete account, however, requires attention not only to maladaptive processes but also to positive body image. Body appreciation reflects respect, acceptance, and care toward the body, including the capacity to value bodily functionality and uniqueness despite sociocultural pressure (Shi et al., 2024; Tiggemann and Slater, 2013). Positive body image is conceptually distinct from merely low body dissatisfaction; rather, it functions as a psychological resource associated with healthier self-regulation, more adaptive motivation for physical activity, and greater comfort in embodied experience. Under conditions of heightened body shame, body appreciation may be diminished (Marta-Simões et al., 2016), thereby reducing access to a self-affirming lens through which physical capability is interpreted. In turn, lower body appreciation may weaken perceived physical ability by reducing readiness to notice competence-related evidence, such as skill improvement or stamina gains, and by increasing threat sensitivity in performance contexts (Kabak et al., 2026).

Existing research has often treated social media exposure as a matter of time spent online (Woodward et al., 2025), platform frequency (Clayborne et al., 2025), or general use intensity (Huang et al., 2025). Such indicators may obscure the psychological mechanism most likely to account for downstream effects: the extent to which social media engagement is organized around comparison as a habitual evaluative mode (Meier and Reinecke, 2021). Social comparison orientation tailored to social networking contexts provides a more precise predictor for capability-related outcomes because it captures the tendency to scan for rank-relevant cues and interpret online information through evaluative frames (Burnell et al., 2024). Another limitation concerns the fragmentation of body image scholarship across negative and positive constructs. Studies have frequently examined shame, surveillance, dissatisfaction, or appreciation in isolation (Piran et al., 2019), leaving uncertainty about how negative self-conscious emotions may cascade into diminished positive body image and subsequently translate into capability beliefs. Physical self-efficacy, particularly perceived physical ability (Ryckman et al., 1982), has rarely been positioned at the end of such a sequential process, even though this construct occupies a central role in behavior change and performance-related adjustment.

A focus on college students further enhances the practical relevance of this model. Interventions on university campuses commonly target exercise participation, mental health, and body image concerns, yet social media comparison dynamics remain difficult to translate into actionable intervention targets without a clearer account of the underlying mechanisms. Identifying body shame and body appreciation as linked processes may suggest leverage points for prevention programs and psychoeducational efforts, including media literacy approaches that reduce evaluative comparison, emotion-focused strategies that mitigate shame responses, and positive body image cultivation that supports embodied confidence.

Accordingly, the present study aimed to examine the association between social media social comparison orientation and physical self-efficacy among Chinese college students, and to test whether body shame and body appreciation mediate this association both independently and sequentially. The present study therefore tested the proposed model in a large sample of Chinese college students and examined whether the comparison–body image–capability linkage identified in the broader literature would also be supported in this culturally specific social media context. Specifically, this study investigated whether higher social media social comparison orientation would be associated with greater body shame and lower body appreciation, whether body shame would be negatively associated with body appreciation and physical self-efficacy, whether body appreciation would be positively associated with physical self-efficacy, and whether body shame and body appreciation would jointly form a sequential pathway linking social media social comparison orientation to physical self-efficacy.

## 2 Literature review and hypotheses

### 2.1 Social media social comparison orientation and capability-related self-evaluations

Social comparison theory conceptualizes self-evaluation as a relational process in which information about others becomes a reference point when objective standards are ambiguous or when self-relevant uncertainty is salient (Festinger, 1954). Individual differences in comparison orientation capture the extent to which comparison is chronically salient and routinely used to interpret social information (Gibbons and Buunk, 1999). In social networking environments, comparison cues are unusually concentrated and repeatedly accessible. Curated self-presentation, visual dominance of body-related content, quantified feedback (e.g., likes), and algorithmic prioritization of popular posts collectively shape a context in which perceived standing can become a prominent interpretive dimension of browsing. This logic may be particularly relevant in the Chinese college context, where social media use is deeply embedded in everyday peer interaction and self-presentation, and where highly visual platform environments may further amplify the salience of appearance- and competence-related comparison cues.

Physical self-efficacy, operationalized as perceived physical ability (Ryckman et al., 1982), reflects confidence in one's physical competence. Capability beliefs are sensitive to evaluative climates because such climates shape how ability-relevant information is selected (Hollenbaugh and Ferris, 2015), interpreted, and weighted. A comparison-oriented mode of social media engagement is therefore expected to align with less favorable capability appraisals, particularly in domains where idealized standards are salient and upward comparison opportunities are frequent. This expectation concerns association patterns rather than temporal ordering.

H1: Social media social comparison orientation is negatively associated with physical self-efficacy.

### 2.2 Social media comparison orientation and body shame

Objectification theory highlights how sociocultural contexts that emphasize appearance evaluation are linked to self-objectification processes, including habitual monitoring of the body and the internalization of external standards (Fredrickson and Roberts, 1997). Body shame represents a self-conscious emotion closely tied to perceived discrepancies from internalized appearance ideals and to self-directed blame. In social media environments, comparison cues are abundant, visually salient, and often centered on idealized or highly curated appearance-related content, making discrepancy-focused self-appraisal especially likely among individuals who are more strongly oriented toward social comparison. Prior research has shown that appearance-related comparison on social networking sites is associated with body image disturbance through objectification-related processes (Seekis et al., 2020), and recent evidence further indicates that appearance comparison on social networking sites is positively associated with body shame (Wang et al., 2025). These findings are consistent with broader work suggesting that social media comparison may heighten vulnerability to shame by increasing perceived distance from internalized ideals and amplifying self-critical evaluation (Bessenoff and Snow, 2006; Kidd et al., 2024; Kvardova et al., 2025). This expectation may be especially meaningful in Chinese college populations, where prior research has shown that media- and peer-related appearance pressures are associated with negative body image through comparison-based processes (Shen et al., 2022). Accordingly, social media social comparison orientation is expected to show a positive association with body shame.

H2: Social media social comparison orientation is positively associated with body shame.

### 2.3 Body shame and physical self-efficacy

Shame is characterized by global negative self-evaluation, heightened evaluative threat sensitivity, and motivations to withdraw or conceal (Castonguay et al., 2016). In physical and exercise-related settings, these characteristics are compatible with lower confidence in bodily competence, because shame tends to foreground perceived deficiencies and increase attentional focus on potential failure or negative evaluation. Empirical work in body image and sport/exercise psychology also indicates that shame-related experiences are typically linked to avoidance orientations, reduced comfort in embodied contexts, and poorer self-evaluations in physical domains (Dimas et al., 2021; Lucibello et al., 2025). From a self-evaluative perspective, body shame may therefore undermine physical self-efficacy by biasing judgments toward inadequacy and by weakening confidence in one's ability to perform effectively in body-salient contexts. These considerations support a negative association between body shame and perceived physical ability.

H3: Body shame is negatively associated with physical self-efficacy.

### 2.4 Social media comparison orientation and body appreciation

Positive body image research conceptualizes body appreciation as a distinct construct involving respect, acceptance, and care for the body, including valuing bodily functionality alongside appearance (Tylka and Wood-Barcalow, 2015b). Social media social comparison orientation is anchored in rank-relevant processing and evaluative attention, which may be less compatible with non-contingent body valuation. When individuals habitually approach social media through comparison, they may become more likely to assess their bodies in relation to idealized others and less likely to adopt an appreciative stance grounded in respect, acceptance, and functionality. Prior work has shown that social comparison is negatively related to body appreciation (Homan and Lemmon, 2015), and more recent evidence suggests that body appreciation plays an important role in the psychological consequences of appearance-based social comparison among social media users (Primo-Simões et al., 2025). In environments saturated with appearance standards and peer display, comparison-oriented engagement is therefore expected to align with lower body appreciation. In the Chinese social media context, where body-related peer communication and appearance-focused online interaction have also been linked to body dissatisfaction among college students, this negative association may be especially relevant to understanding how comparison-oriented browsing undermines positive body image (Wang et al., 2022).

H4: Social media social comparison orientation is negatively associated with body appreciation.

### 2.5 Body appreciation and physical self-efficacy

Self-efficacy theory describes efficacy beliefs as judgments about capability that are linked to motivation, persistence, and adaptive self-regulation (Bandura et al., 1997). Body appreciation supports an embodied stance characterized by acceptance and a functionality focus, which tends to align with more adaptive interpretations of bodily sensations, greater comfort in physical activity settings, and more constructive engagement with capability-relevant feedback. In exercise and health psychology, positive body image indicators are consistently associated with more favorable exercise-related cognitions and higher perceived competence (Jankauskiene et al., 2020, 2024). Related evidence also suggests that body appreciation is positively linked to self-evaluative resources relevant to efficacy beliefs; for example, (El-Sayed et al. 2024) reported that body appreciation was positively associated with self-efficacy among community-dwelling older women. Although that study focused on a different population, it supports the broader proposition that a more appreciative and accepting relationship with the body may foster stronger confidence in one's physical capability. These patterns support a positive association between body appreciation and perceived physical ability.

H5: Body appreciation is positively associated with physical self-efficacy.

### 2.6 Indirect associations: single mediators and sequential (chain) mediation

The conceptual integration of social comparison theory, objectification theory, and positive body image perspectives implies that social media social comparison orientation may relate to physical self-efficacy through multiple, partially overlapping pathways. Two single-mediator indirect associations are theoretically coherent. One pathway involves body shame: comparison-oriented engagement aligns with discrepancy-focused appraisal and self-conscious distress, and recent work on appearance comparison in social networking contexts directly supports the relevance of this link (Wang et al., 2025). Body shame, in turn, is theoretically and empirically compatible with less favorable capability beliefs in physical domains. A second pathway involves body appreciation: comparison-oriented engagement is likely to undermine non-contingent body valuation, and prior work suggests that social comparison is negatively associated with body appreciation, while body appreciation functions as a meaningful protective factor in the context of appearance-based comparison (Homan and Lemmon, 2015; Primo-Simões et al., 2025). Body appreciation, in turn, aligns with more favorable perceived physical ability. In addition, prior findings from Chinese samples suggest that comparison-based body image processes may involve interconnected vulnerability and protective mechanisms rather than a single negative outcome. This broader pattern is consistent with examining body shame and body appreciation not only as separate mediators but also as a sequential body-related pathway in the present study.

Beyond these distinct indirect associations, the two mediators may also form a sequential linkage. Body shame and body appreciation represent contrasting modes of body-related self-relation, and prior work commonly reports an inverse association between shame-related body experiences and positive body image. In such a configuration, comparison orientation aligns with higher body shame, body shame aligns with lower body appreciation, and lower body appreciation aligns with lower perceived physical ability. This sequential arrangement is theoretically meaningful because it captures how a comparison-based, discrepancy-focused mode of social media engagement may first heighten body-related self-conscious distress and then erode positive body valuation, thereby corresponding to lower physical self-efficacy. This chain indirect association is consistent with a model that remains agnostic about temporal ordering while specifying a coherent body-related explanatory process.

H6: Body shame mediates the association between social media social comparison orientation and physical self-efficacy.
H7: Body appreciation mediates the association between social media social comparison orientation and physical self-efficacy.
H8: Body shame and body appreciation sequentially mediate the association between social media social comparison orientation and physical self-efficacy, such that higher comparison orientation is associated with higher body shame, higher body shame is associated with lower body appreciation, and lower body appreciation is associated with lower physical self-efficacy.

**Figure 1:**
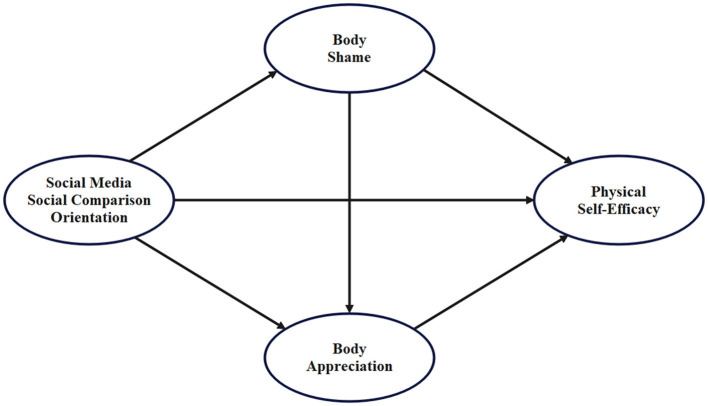
Hypothesized model.

The hypothesized direct association, the two specific indirect associations, and the sequential indirect association are summarized in Figure 1.

## 3 Materials and methods

### 3.1 Participants

This cross-sectional study was conducted in China, specifically in Shandong Province, among undergraduate students from 12 universities: Shandong University of Technology, Shandong Normal University, Ocean University of China, China University of Petroleum, Shandong University, Qingdao University, Shandong Sport University, Shandong Agricultural University, Shandong University of Science and Technology, Qingdao University of Science and Technology, Linyi University, and Liaocheng University. Data were collected between October 2025 and December 2025 using an anonymous online survey administered through Wenjuanxing. Participant recruitment was coordinated with the assistance of local collaborators in Shandong Province, China. After obtaining permission from the relevant university units or contact persons at each participating institution, designated local contacts disseminated the survey link through university-affiliated online channels, including class communication groups, university social media groups, student organization networks, and learning platforms. The authors were responsible for the study design, questionnaire administration, and data analysis, whereas the role of the local collaborators was limited to distributing the survey invitation. No financial compensation was provided to the local collaborators or participating institutions for survey dissemination.

A non-probability convenience sampling strategy was used. Therefore, the sample should not be considered fully representative of all college students in China, and the generalizability of the findings should be interpreted with caution. Before accessing the questionnaire, participants were presented with an online information page describing the purpose of the study, the voluntary and anonymous nature of participation, the approximate time required to complete the survey, and their right to withdraw at any time without penalty. Participants were informed that completion and submission of the questionnaire would be taken as implied informed consent. The study protocol was reviewed and approved by the Institutional Review Board of Pai Chai University, South Korea.

A total of 3,569 questionnaires were returned. The online survey platform was configured with a forced-response setting, such that questionnaires could not be submitted with unanswered items. Accordingly, there were no item-level missing data among the returned questionnaires. Data screening followed predefined quality-control procedures for online surveys. Cases were excluded if they met at least one of the following criteria: (a) uniform or patterned responding (e.g., straight-lining across Likert-type items); (b) logically inconsistent responses to demographic questions; or (c) an implausibly short completion time indicating insufficient engagement. After removing invalid responses, 3,401 valid questionnaires were retained, yielding an effective response rate of 95.29%.

For transparency, the participant flow was as follows: 3,569 questionnaires were returned, 168 were excluded during data screening, and 3,401 were included in the final analyses. Demographic characteristics of the participants are presented in Table 1, including gender, age, year of study, major type, BMI category, weekly exercise frequency, and daily social media use duration. The final sample size was considered more than adequate for the planned analyses. The full questionnaire included 39 items across all study measures, and the effective sample of 3,401 substantially exceeded commonly cited heuristic recommendations for sample size adequacy in factor analysis and structural equation modeling (Jackson, 2003; Tinsley and Tinsley, 1987). This sample size therefore provided a strong basis for robust parameter estimation and model stability in confirmatory factor analysis and structural equation modeling.

**Table 1:** Demographic characteristics of participants (*N* = 3,401).

Variable	Category	*n*	%
Gender	Male	1,428	41.99
	Female	1,973	58.01
Age	17–18	612	17.99
	19–20	1,214	35.70
	21–22	1,020	29.99
	≥23	555	16.32
	Year of study	Freshman	874	25.70
Sophomore		843	24.79
Junior		852	25.05
Senior		832	24.46
Major type	Humanities and social sciences	1,186	34.87
	Science and engineering	1,463	43.02
	Arts and sports	752	22.11
BMI category	Underweight	524	15.41
	Normal weight	2,256	66.33
	Overweight	431	12.67
	Obese	190	5.59
Weekly exercise frequency	≤ 1 time	1,023	30.08
	2–3 times	1,448	42.58
	≥4 times	930	27.34
Daily social media use	< 1 h	487	14.32
	1–2 h	1,187	34.90
	3–4 h	1,098	32.28
	≥5 h	629	18.49

### 3.2 Measurement tools

All instruments were administered in Chinese. For the Iowa–Netherlands Comparison Orientation Measure (INCOM), the Body Shame subscale of the Objectified Body Consciousness Scale (OBCS), the Body Appreciation Scale−2 (BAS-2), and the Perceived Physical Ability (PPA) subscale of the Physical Self-Efficacy Scale, previously translated, revised, or validated Chinese versions were used. To enhance response consistency across measures in the present survey, all items were presented using a unified 5-point Likert response format. Higher scores indicated higher levels of the corresponding construct after reverse coding where necessary. Internal consistency reliability was assessed using Cronbach's alpha. The use of Chinese versions of these instruments was supported by prior psychometric or revision studies in Chinese samples.

Social media social comparison orientation was assessed using the social networking site–adapted version of the Iowa–Netherlands Comparison Orientation Measure (INCOM), following Yang's (2016) social media adaptation and using the validated Chinese version of the INCOM as the language basis (Wang et al., 2006). The scale captures individual differences in comparison orientation in social media contexts, including ability- and opinion-based comparison. A representative item is: “I always pay a lot of attention to how I do things compared with how others do things.” All items were rated on a 5-point Likert scale ranging from 1 (strongly disagree) to 5 (strongly agree). Items designated as reverse-scored in the original instrument were recoded prior to analysis so that higher scores reflected stronger social media social comparison orientation. Cronbach's α in the present sample was 0.888, indicating good internal consistency. The use of the Chinese INCOM is supported by prior validation work in Chinese samples.

Body shame was measured using the Body Shame subscale of the Objectified Body Consciousness Scale (OBCS) (McKinley and Hyde, 1996). In the present study, the Chinese revised version for college students was used, following the revision reported by (Chen and Jiang 2007). This subscale assesses self-conscious emotional responses related to perceived discrepancies from internalized body standards. A representative item is: “When I can't control my weight, I feel like something must be wrong with me.” Items were rated on a 5-point Likert scale ranging from 1 (strongly disagree) to 5 (strongly agree). Reverse-scored items were recoded prior to computing the composite score so that higher values represented higher levels of body shame. The scale demonstrated excellent internal consistency in this study (Cronbach's α = 0.919). Prior Chinese research has reported acceptable psychometric performance of the revised OBCS in Chinese undergraduate samples.

Body appreciation was assessed using the Chinese version of the Body Appreciation Scale−2 (BAS-2) (Tylka and Wood-Barcalow, 2015a). Given the Chinese college student context of the present study, we used the Mandarin Chinese version validated in Chinese mainland samples and note that the BAS-2 has also shown satisfactory psychometric properties in a Standard Chinese version. A representative item is: “I feel good about my body.” Participants responded on a 5-point scale ranging from 1 (never) to 5 (always). After reverse coding according to scale instructions, higher scores indicated greater body appreciation. The scale showed excellent reliability in the current sample (Cronbach's α = 0.928). This choice is supported by prior evidence demonstrating satisfactory psychometric properties of Chinese BAS-2 versions, as well as broader cross-national evidence for measurement invariance of the BAS-2 across 65 nations and 40 languages (Ma et al., 2022; Swami et al., 2023).

Physical self-efficacy was operationalized using the Perceived Physical Ability (PPA) subscale of the Physical Self-Efficacy Scale (Ryckman et al., 1982). In the present study, the Chinese revised version reported by (Sun et al. 2005) was used. This subscale assesses confidence in physical competence and capability. A representative item is: “I have excellent reflexes.” All items were rated on a 5-point Likert scale ranging from 1 (strongly disagree) to 5 (strongly agree). Reverse-scored items were transformed prior to analysis to ensure that higher scores reflected stronger perceived physical ability. In the present study, Cronbach's α was 0.921, indicating excellent internal consistency. Prior Chinese research has supported the use of the revised Physical Self-Efficacy Scale in college student samples.

### 3.3 Data analysis

All statistical analyses were conducted using IBM SPSS Statistics 26.0 and AMOS 26.0. Prior to hypothesis testing, preliminary data screening was performed to evaluate distributional characteristics and potential violations of normality assumptions. Invalid questionnaires had already been excluded during the data-cleaning stage. Skewness and kurtosis values for all observed variables fell within recommended thresholds (absolute skewness < 2, absolute kurtosis < 7), supporting the use of maximum likelihood estimation in confirmatory factor analysis and structural equation modeling (Curran et al., 1996).

Descriptive statistics were calculated for social media social comparison orientation, body shame, body appreciation, and physical self-efficacy. Internal consistency reliability was assessed using Cronbach's α. Composite reliability (CR) and average variance extracted (AVE) were computed to evaluate construct reliability and convergent validity. CR values above 0.70 and AVE values above 0.50 were considered acceptable (Fornell and Larcker, 1981). Standardized factor loadings above 0.50, and preferably above 0.70, were regarded as satisfactory (Hair et al., 2018).

Confirmatory factor analysis (CFA) was conducted to examine the measurement model comprising four latent variables: social media social comparison orientation, body shame, body appreciation, and physical self-efficacy. Model fit was evaluated using multiple indices, including the chi-square to degrees of freedom ratio (χ^2^/df), Comparative Fit Index (CFI), Tucker–Lewis Index (TLI), Standardized Root Mean Square Residual (SRMR), and Root Mean Square Error of Approximation (RMSEA) with its 90% confidence interval. The following criteria were adopted to determine acceptable fit: χ^2^/df < 5.00, CFI and TLI ≥ 0.90 for acceptable fit and ≥ 0.95 for good fit, SRMR < 0.08, and RMSEA < 0.08 for acceptable fit and < 0.05 for close fit (Browne and Cudeck, 1992; Hu and Bentler, 1999). To further examine discriminant validity, alternative models (one-factor, two-factor, and three-factor structures) were compared with the hypothesized four-factor model.

Given that all variables were measured via self-report, common method bias was assessed. Harman's single-factor test was conducted using unrotated exploratory factor analysis to determine whether a single factor accounted for the majority of variance. Common method variance was considered unlikely to substantially influence results if the first unrotated factor explained less than 40% of total variance (Podsakoff et al., 2003). Additionally, the fit of the hypothesized four-factor measurement model was compared with that of a single-factor model in CFA; substantially poorer fit for the single-factor model provided further evidence against serious common method bias (Williams et al., 2010).

Pearson correlation analyses were performed to examine bivariate associations among the four constructs. The hypothesized mediation model was tested using structural equation modeling in AMOS 26.0. The primary aim of the study was to test the hypothesized psychological mechanism linking social media social comparison orientation to physical self-efficacy through body shame and body appreciation. Therefore, the structural model focused on the theoretically specified associations among the focal constructs rather than including demographic and behavioral covariates in the main model. The structural model specified direct paths from social media social comparison orientation to physical self-efficacy, as well as indirect paths through body shame and body appreciation, including both specific indirect effects (via each mediator separately) and a sequential indirect effect (social media social comparison orientation → body shame → body appreciation → physical self-efficacy). Model fit was evaluated using the same criteria described above. Indirect effects were examined using bias-corrected bootstrapping with 5,000 resamples. Mediation effects were considered statistically significant when the 95% confidence interval did not include zero (Preacher and Hayes, 2008).

## 4 Results

### 4.1 Preliminary analyses and measurement properties

Descriptive statistics and reliability indicators for all study variables are presented in Table 2. Social media social comparison orientation showed a mean below the midpoint of the scale, whereas body appreciation and physical self-efficacy were above the midpoint. Body shame was at a moderate level. Internal consistency coefficients (Cronbach's α) ranged from 0.888 to 0.928, indicating good to excellent reliability across all constructs.

**Table 2:** Descriptive statistics and measurement properties of study variables (*N* = 3,401).

Variable	Mean	SD	α	Factor loading	CR	AVE
Social media social comparison orientation	2.35	0.74	0.888	0.678–0.864	0.750	0.603
Body shame	2.56	0.97	0.919	0.723–0.814	0.919	0.588
Body appreciation	3.45	0.92	0.928	0.721–0.773	0.928	0.565
Physical self-efficacy	3.72	0.85	0.921	0.719–0.753	0.922	0.541

SD, standard deviation; α, Cronbach's alpha; CR, composite reliability; AVE, average variance extracted.

Results from confirmatory factor analysis further supported the adequacy of the measurement model. Standardized factor loadings were all within acceptable ranges and exceeded recommended thresholds. Composite reliability values were above 0.70 for all constructs, and average variance extracted values exceeded 0.50, supporting convergent validity. Overall, the measurement properties of the four latent variables met established psychometric criteria, providing a sound basis for subsequent structural analyses.

### 4.2 Common method bias test

Given that all variables were assessed using self-report questionnaires, potential common method bias was examined using both exploratory and confirmatory approaches. Harman's single-factor test was first conducted through unrotated exploratory factor analysis. The first unrotated factor accounted for less than 40% of the total variance, suggesting that common method variance did not dominate the data structure.

To further assess potential method bias, a series of confirmatory factor analyses were performed to compare alternative measurement models. As shown in Table 3, the hypothesized four-factor model demonstrated substantially better fit than the one-factor, two-factor, and three-factor models. The one-factor model exhibited very poor fit (χ^2^/df = 72.234, CFI = 0.499, RMSEA = 0.145), indicating that the covariance among items could not be adequately explained by a single latent construct. Model fit improved progressively as additional latent factors were specified, with the four-factor model showing good fit indices (χ^2^/df = 6.077, CFI = 0.965, TLI = 0.962, SRMR = 0.020, RMSEA = 0.039). An unmeasured latent method construct (ULMC) model was also tested by adding a latent common method factor to the four-factor model. The fit indices of the ULMC-factor model were nearly identical to those of the original four-factor model, and no meaningful improvement in model fit was observed. These findings indicate that common method bias was unlikely to substantially influence the results.

**Table 3:** Comparison of alternative measurement models for common method bias (*N* = 3,401).

Model	χ^2^/df	CFI	TLI	SRMR	RMSEA (90% CI)
One-factor	72.234	0.499	0.462	–	0.145 (0.143, 0.146)
Two-factor	41.179	0.718	0.696	0.120	0.109 (0.107, 0.110)
Three-factor	9.015	0.944	0.939	0.050	0.049 (0.047, 0.050)
Four-factor	6.077	0.965	0.962	0.020	0.039 (0.037, 0.040)
ULMC-factor	6.093	0.965	0.962	0.020	0.039 (0.037, 0.040)

χ^2^/df, chi-square to degrees of freedom ratio; CFI, Comparative Fit Index; TLI, Tucker–Lewis Index; SRMR, Standardized Root Mean Square Residual; RMSEA, Root Mean Square Error of Approximation; CI, confidence interval; ULMC-factor, unmeasured latent method construct model.

### 4.3 Correlations and discriminant validity

Pearson correlation coefficients among the study variables are presented in Table 4. Social media social comparison orientation was positively correlated with body shame and negatively correlated with both body appreciation and physical self-efficacy. Body shame showed negative associations with body appreciation and physical self-efficacy. Body appreciation was positively associated with physical self-efficacy. All correlations were statistically significant at the *p* < 0.001 level and in directions consistent with the hypothesized model. The magnitudes of the correlations ranged from small to moderate, suggesting related yet distinguishable constructs.

**Table 4:** Correlations and discriminant validity among study variables (*N* = 3,401).

Variable	Social media social comparison orientation	Body shame	Body appreciation	Physical self-efficacy
Social media social comparison orientation	0.777			
Body shame	0.257^***^	0.767		
Body appreciation	−0.277^***^	−0.334^***^	0.752	
Physical self-efficacy	−0.344^***^	−0.339^***^	0.375^***^	0.736

Values on the diagonal represent the square roots of average variance extracted (AVE). Off-diagonal values represent Pearson correlation coefficients.

^***^p < 0.001.

Discriminant validity was further evaluated using the Fornell–Larcker criterion. As shown in Table 4, the square roots of the average variance extracted (displayed on the diagonal) exceeded the corresponding inter-construct correlations, supporting adequate discriminant validity among the four latent variables. These findings indicate that each construct captured unique variance beyond its shared variance with other constructs, providing empirical justification for proceeding with structural model testing.

### 4.4 Structural equation modeling analysis

The hypothesized structural model was tested using structural equation modeling. The overall model demonstrated good fit to the data [χ^2^/df = 6.077, CFI = 0.965, TLI = 0.962, SRMR = 0.020, RMSEA = 0.039, 90% CI (0.037, 0.040)]. Although the χ^2^/df ratio was slightly above conventional heuristic thresholds, chi-square statistics are highly sensitive to large sample sizes and model complexity. Given the substantial sample size (*N* = 3,401) and the number of estimated parameters, the elevated χ^2^/df is not unexpected. Importantly, incremental and residual-based fit indices (CFI, TLI, SRMR, RMSEA) consistently indicated satisfactory to close fit, supporting the adequacy of the structural model.

**Figure 2:**
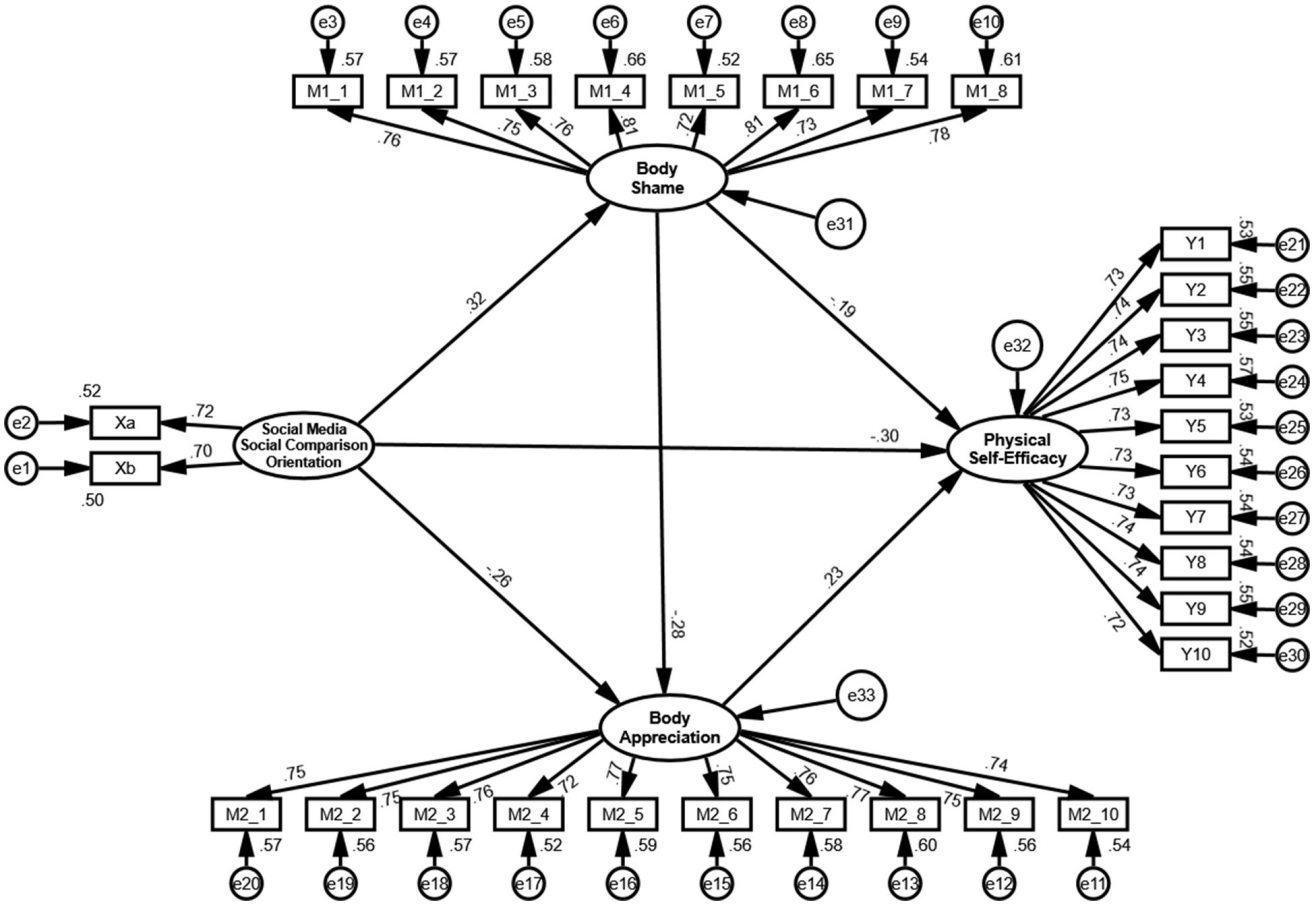
Structural model with standardized path coefficients (*N* = 3,401). Values represent standardized regression coefficients. All paths shown are statistically significant.

Standardized path coefficients are presented in Figure 2. Social media social comparison orientation was negatively associated with physical self-efficacy (β = −0.30), positively associated with body shame (β = 0.32), and negatively associated with body appreciation (β = −0.26). Body shame showed a negative association with physical self-efficacy (β = −0.19) and a negative association with body appreciation (β = −0.28). Body appreciation was positively associated with physical self-efficacy (β = 0.23). All reported paths reached statistical significance (*p* < 0.05). These findings provide empirical support for H1–H5, which concerned the direct associations among the constructs.

### 4.5 Bootstrapping mediation test

To further examine the indirect effects, bias-corrected bootstrapping with 5,000 resamples was conducted. The mediation analysis simultaneously estimated the direct effect, two specific indirect effects (via body shame and body appreciation), and the sequential indirect effect (via body shame and body appreciation in sequence). Mediation was considered statistically significant when the 95% bias-corrected confidence interval did not include zero.

As presented in Table 5, the total effect of social media social comparison orientation on physical self-efficacy was significant [β = −0.441, 95% CI (−0.478, −0.403)]. The direct effect remained significant after accounting for mediators [β = −0.298, 95% CI (−0.342, −0.253)], indicating partial mediation. The total indirect effect was also significant [β = −0.143, 95% CI (−0.163, −0.124)], accounting for 32.43% of the total effect. Regarding specific indirect pathways, the indirect effect through body shame was significant [β = −0.061, 95% CI (−0.076, −0.049)], supporting H6. The indirect effect through body appreciation was likewise significant [β = −0.061, 95% CI (−0.075, −0.048)], supporting H7. In addition, the sequential indirect effect through body shame and body appreciation was significant [β = −0.021, 95% CI (−0.027, −0.016)], supporting H8. The proportions of the total effect accounted for by the two specific indirect effects were equal (13.83% each), whereas the sequential indirect pathway accounted for 4.76% of the total effect.

**Table 5:** Bootstrapping results for direct, specific indirect, and sequential indirect effects (*N* = 3,401).

Path	β	Boot SE	Boot LLCI	Boot ULCI	Ratio (%)
**Direct effect**	−0.298	0.023	−0.342	−0.253	67.57
**Indirect effects**	−0.143	0.010	−0.163	−0.124	32.43
Social media social comparison orientation → body shame → physical self-efficacy	−0.061	0.007	−0.076	−0.049	13.83
Social media social comparison orientation → body appreciation → physical self-efficacy	−0.061	0.007	−0.075	−0.048	13.83
Social media social comparison orientation → body shame → body appreciation → physical self-efficacy	−0.021	0.003	−0.027	−0.016	4.76
**Total effect**	−0.441	0.020	−0.478	−0.403	100

Boot SE, bootstrap standard error; Boot LLCI, lower limit of 95% bias-corrected confidence interval; Boot ULCI, upper limit of 95% bias-corrected confidence interval. Ratio (%) represents the proportion of each effect relative to the total effect.

Taken together, these findings indicate that body shame and body appreciation both function as mediating mechanisms in the association between social media social comparison orientation and physical self-efficacy, operating both independently and in sequence. The persistence of a significant direct effect suggests that additional pathways beyond the current mediators may also contribute to the overall association.

## 5 Discussion

### 5.1 Summary of key findings

The present study examined the associations between social media social comparison orientation and physical self-efficacy among Chinese college students, with body shame and body appreciation specified as two mediating mechanisms, both independently and sequentially. Results indicated a robust negative association between social media comparison orientation and physical self-efficacy. Social media comparison orientation was also associated with higher body shame and lower body appreciation; body shame and body appreciation were, in turn, associated with lower and higher physical self-efficacy, respectively. Bootstrapping estimates further suggested that the overall association between comparison orientation and physical self-efficacy was statistically linked to three indirect pathways: a specific indirect association via body shame, a specific indirect association via body appreciation, and a sequential indirect association through body shame and body appreciation. Together, these findings map a coherent set of body-related psychological correlates that accompany social media comparison orientation in relation to capability beliefs. In addition, because these associations were observed among Chinese college students, the findings provide context-specific evidence that the comparison–body image–capability linkage is also relevant in a non-Western university setting.

### 5.2 Theoretical implications

#### 5.2.1 Social media comparison orientation and capability beliefs

Prior research on social media comparison has largely concentrated on affective and evaluative outcomes such as body dissatisfaction, negative affect, depressive symptoms, and eating-related pathology. By focusing on physical self-efficacy—operationalized as perceived physical ability—the present study extends social comparison scholarship to a capability-centered construct with direct relevance to health behavior, persistence, and performance-oriented functioning. Importantly, the focal predictor in the present study was not social media exposure intensity per se, but comparison orientation within social media contexts. This distinction is theoretically meaningful because comparison orientation reflects a relatively stable evaluative processing tendency: the same platform exposure may be psychologically neutral for some users, yet highly self-relevant for individuals who habitually scan for relative standing. Locating capability beliefs within this evaluative processing framework helps explain why social media experiences may be associated with differences in perceived competence even when objective physical functioning is not directly assessed (Bandura, 1986; Burnell et al., 2024).

This interpretation is broadly consistent with recent review-level evidence showing that social media-related comparison is systematically associated with body image concerns, eating-related symptoms, and broader indicators of psychological well-being (Behera et al., 2025; Bonfanti et al., 2025; Sarda et al., 2025). At the same time, these reviews also suggest that the consequences of social media use are not uniform across platforms, user groups, or cultural settings. Platform affordances differ in the extent to which they foreground appearance, fitness, lifestyle display, or social feedback, and these differences may shape the salience of comparison cues. Likewise, findings from adolescents, young adults, and older populations suggest that the psychological meaning of social media comparison may vary across developmental stages and sociocultural contexts (Clayborne et al., 2025; Huang et al., 2025; Woodward et al., 2025). In that sense, the present findings contribute to a broader international literature by showing that, among Chinese college students, comparison-oriented processing on social media is also linked to lower capability beliefs. This extends the discussion beyond body dissatisfaction and negative mood to perceived physical ability, and it suggests that capability beliefs may be embedded in the same social-cognitive comparison processes highlighted in contemporary international research. At the same time, the present results should not be read as implying full equivalence across cultural contexts; rather, they indicate that this capability-related association is also observable within the contemporary Chinese college social media environment.

#### 5.2.2 Integrating objectification-relevant affect with positive body image

A second contribution lies in linking a self-conscious emotion emphasized in objectification theory—body shame—with a protective construct from the positive body image tradition—body appreciation. Much of the empirical literature has examined negative and positive body-related constructs in parallel rather than in the same explanatory model (Burychka et al., 2021; Lucibello et al., 2023; Tylka and Piran, 2019; Tylka and Wood-Barcalow, 2015b). The current findings support the value of integrating these literatures: body shame and body appreciation showed distinct associations with physical self-efficacy and carried separate indirect associations between social media comparison orientation and capability beliefs.

The body shame pathway is consistent with prior research indicating that appearance-related comparison in social networking environments may foster body-related self-conscious distress through discrepancy-focused self-evaluation and objectification-relevant processes (Seekis et al., 2020; Wang et al., 2025). In highly visual and appearance-salient online contexts, comparison may make perceived inadequacies more psychologically accessible, thereby heightening shame and undermining confidence in bodily competence. At the same time, the pathway through body appreciation highlights that comparison processes are not only linked to vulnerability, but may also erode protective forms of body-related self-regard. This interpretation is in line with evidence that social comparison is negatively related to body appreciation (Homan and Lemmon, 2015), and with more recent findings indicating that body appreciation remains theoretically important in the psychological consequences of appearance-based social comparison among social media users (Primo-Simões et al., 2025).

Taken together, these findings suggest that negative evaluative affect and positive body-related valuation are not interchangeable indicators on a single continuum (Liang et al., 2021; Marta-Simões et al., 2016; More et al., 2024; Raspovic et al., 2023). Rather, they appear to represent partly distinct psychological correlates through which social media comparison orientation is linked to perceived physical ability. From a broader international perspective, this interpretation also resonates with contemporary systematic reviews emphasizing that body image-related outcomes in social media settings involve not only self-objectification and body concerns, but also self-compassion and other protective self-relational resources (Sarda et al., 2025). The present findings therefore support a more differentiated account of body-related self-evaluation in social media environments—one that accommodates both vulnerability processes (shame-linked self-devaluation) and resource processes (appreciation-linked embodied confidence) (Couto and Willoughby, 2024; Marta-Simões et al., 2016; Mills et al., 2022; Yao et al., 2021). In the present Chinese college sample, this integrated pattern may be especially informative because peer interaction, appearance visibility, and self-presentation are tightly interwoven in students' everyday social media use.

#### 5.2.3 A sequential body-related linkage associated with capability beliefs

Beyond the two specific indirect pathways, the sequential indirect association through body shame and body appreciation adds nuance to how body-related processes may cluster around social media comparison orientation. Body shame was inversely associated with body appreciation, and the chain pathway was statistically supported in addition to the single-mediator pathways. Conceptually, this sequential pattern is consistent with the notion that self-conscious distress about the body may coexist with, and potentially undermine, a more accepting and caring stance toward the body (Liang et al., 2021), which in turn corresponds to less favorable capability beliefs. In this sense, the results help clarify how comparison-oriented engagement on social media may be linked not only to isolated negative reactions, but to a broader self-evaluative configuration in which heightened shame and reduced appreciation jointly correspond to lower perceived physical ability.

This interpretation is also consistent with emerging work suggesting that body appreciation may serve as an important psychological buffer within comparison-based processes, even though its protective role may vary across populations and settings (Primo-Simões et al., 2025). Likewise, findings from non-college or older samples suggest that body appreciation may be positively associated with self-efficacy-related outcomes more generally (El-Sayed et al., 2024), which lends indirect support to the idea that diminished appreciation may weaken confidence in one's physical capability. Although the sequential component accounted for a smaller share of the total association than the specific indirect pathways, its primary value is explanatory: it captures an interlocking pattern in which negative body-related affect and diminished positive body image co-occur and jointly correspond to lower perceived physical ability. The findings therefore encourage models that treat positive body image as an active psychological resource embedded within broader body-related self-evaluation systems, rather than as a mere correlate examined in isolation (Mills et al., 2022). More broadly, future research may benefit from examining whether the relative strength of shame-based and appreciation-based pathways differs across social media ecologies, age groups, and cultural contexts.

### 5.3 Practical implications

The findings have several implications for interventions targeting college students' well-being and physical functioning beliefs in social media-saturated environments. First, campus-based media literacy initiatives may benefit from moving beyond generic “screen time” messaging to address comparison-oriented processing (Andrade et al., 2023). Recent international review evidence suggests that the harms associated with social media use are shaped not only by quantity of use, but also by the kinds of self-evaluative and body-related processes that online environments activate (Behera et al., 2025; Bonfanti et al., 2025; Sarda et al., 2025). Training that helps students recognize rank-focused interpretations, reduce automatic upward comparison, and diversify self-relevant standards may therefore be more closely aligned with the psychological mechanism implicated by the present results. Content-level strategies—such as deliberately curating feeds away from highly idealized physique content and toward function-oriented or skill-learning content—may also help weaken evaluative salience during browsing (Golmohamadi et al., 2025; Zeng et al., 2021). Such strategies may be especially important because different platforms may vary in the degree to which they emphasize appearance display, fitness aspiration, or social feedback.

Second, body image interventions may gain leverage by addressing both body shame reduction and body appreciation enhancement. Shame-focused components can emphasize emotion regulation and self-compassion practices that target self-blame and perceived defectiveness in response to appearance-related cues. This is consistent with prior work suggesting that comparison-based body distress in social media environments is closely tied to shame-related and objectification-relevant processes (Seekis et al., 2020; Wang et al., 2025). In parallel, interventions that cultivate body appreciation—such as functionality-focused writing, gratitude toward bodily capacities, and acceptance-based exercises—may support a more adaptive embodied stance (Zeng et al., 2021). The current pattern of results suggests that these two targets are not redundant; each aligns with unique variance in physical self-efficacy and with distinct indirect associations from social media comparison orientation. In this sense, prevention and intervention programs may benefit from balancing risk reduction with the active cultivation of positive body image resources.

Third, programs designed to promote physical activity and confidence in physical competence in college settings may benefit from integrating body image content into activity-based interventions. For students high in comparison orientation, discomfort in evaluative exercise environments can coincide with lower perceived physical ability. Structuring physical activity opportunities to reduce appearance evaluation—for example, by emphasizing mastery goals, skill progression, and private feedback—may help sustain participation and support capability beliefs. More broadly, strengthening positive body image may provide a psychologically supportive context in which students interpret physical effort and performance feedback in ways that reinforce confidence rather than undermine it (Yuan et al., 2025). Given international evidence that the psychological effects of social media may vary across platforms, countries, and age groups, intervention design may also need to be adapted to local media ecologies and cultural norms rather than assuming that one approach will generalize equally well across settings. In the present Chinese college context, this may be especially relevant because social media is deeply integrated into students' everyday communication and self-presentation, making comparison-oriented processing a potentially important target for campus mental health and health-promotion efforts.

### 5.4 Limitations

Several limitations should be considered when interpreting the present findings. First, the study relied on a cross-sectional design; accordingly, the reported associations and indirect pathways reflect statistical relations within a single measurement occasion rather than temporal dynamics. Second, all constructs were assessed via self-report instruments, which may be influenced by shared method variance and response tendencies, despite the diagnostic evidence suggesting that common method bias was unlikely to account for the observed factor structure and model fit. Third, participants were recruited from 12 universities in Shandong Province, China, through university-affiliated online channels using convenience sampling. Accordingly, the sample should not be regarded as fully representative of all college students in China, and the findings should be generalized to other institutional, regional, or cultural contexts with caution. In particular, because the study was conducted within a specific Chinese social media and university context, the magnitude and configuration of the observed associations should not be assumed to generalize directly to other national, cultural, or institutional settings. Fourth, although several demographic and behavioral characteristics, including gender, BMI, weekly exercise frequency, and daily social media use duration, were assessed, these variables were not included as covariates in the main structural model. Because such factors may be associated with both social media comparison processes and physical self-efficacy, residual confounding cannot be ruled out. The primary aim of the present study was to test the theoretically specified psychological mechanism linking social media social comparison orientation to physical self-efficacy through body shame and body appreciation; however, future research should examine the proposed model with more comprehensive covariate adjustment. Fifth, the measures indexed broad constructs (e.g., social media comparison orientation) rather than platform-specific behaviors or content types; different platforms and comparison domains (appearance vs. performance vs. lifestyle) may show heterogeneous association patterns. Finally, physical self-efficacy was operationalized as perceived physical ability; although theoretically central, it remains a subjective capability belief and was not paired with objective indicators of physical performance or activity.

### 5.5 Directions for future research

Future research may extend the present work in several ways. Longitudinal designs could clarify whether social media comparison orientation, body shame, body appreciation, and physical self-efficacy exhibit stable reciprocal relations across time and whether indirect pathways persist when prior levels of each construct are controlled. Experimental or quasi-experimental approaches could complement correlational evidence by manipulating comparison exposure (e.g., upward vs. neutral content, appearance- vs. ability-focused posts) to examine short-term shifts in body-related affect, positive body image, and perceived capability. Additional construct refinement is also warranted. Separating ability-based and opinion-based comparison facets within social media contexts, and distinguishing comparison targets (peers vs. influencers) or comparison domains (appearance, fitness performance, lifestyle), may identify more specific mechanisms. Multi-group analyses could test whether the structural relations are invariant across gender, BMI categories, or exercise frequency strata, which would inform tailored intervention targets. Finally, incorporating objective or behavioral indicators—such as device-based physical activity metrics, fitness test results, or performance tasks—would strengthen inference by linking social-cognitive processes to observable physical functioning.

## 6 Conclusion

This study examined the association between social media social comparison orientation and physical self-efficacy among Chinese college students and tested the mediating roles of body shame and body appreciation. The findings indicated that higher social media social comparison orientation was associated with lower physical self-efficacy, higher body shame, and lower body appreciation. In addition, body shame and body appreciation were each linked to the association between social media social comparison orientation and physical self-efficacy as specific indirect pathways, and they also formed a significant sequential indirect pathway. Overall, the findings suggest that both body-related self-conscious emotion and positive body image are important for understanding how social media comparison orientation is associated with perceived physical ability in Chinese college students. These results extend current research on social media comparison by highlighting physical self-efficacy as a relevant outcome and by showing, in a Chinese college context, that both vulnerability-related and protective body-image processes should be considered in future research and intervention efforts.

## Data Availability

The original contributions presented in the study are included in the article/supplementary material, further inquiries can be directed to the corresponding author.
